# Development of a new DNA extraction protocol for PFGE typing of *Mycobacterium tuberculosis* complex

**Published:** 2012-03

**Authors:** Ghodousi A Arash, S Vatani, Davood Darban-Sarokhalil, Maryam Omrani, AA Fooladi, AD Khosaravi, Mohammad Mehdi Feizabadi

**Affiliations:** 1 Department of Microbiology, School of Medicine, Tehran University of Medical Sciences, Tehran, Iran; 2 ADept. of Microbiology, School of Medicine & Infectious and Tropical Diseases Research Centre, Ahvaz Jundishapur University of Medical Sciences, Ahvaz, Iran; 3 Research Center of Molecular Biology, Department of Pathology, School of Medical Sciences, Baqiyatallah University of Medical Sciences, Tehran, Iran

**Keywords:** *Mycobacterium tuberculosis*, TB-complex, Pulsed-filed gel electrophoresis

## Abstract

A modified pulsed-field gel electrophoresis (PFGE) protocol was developed and applied to clinical isolates of Mycobacterium tuberculosis complex to reduce the cost of using lyticase. This protocol reduces the expense of PFGE typing of Mycobacterium tuberculosis complex as it removes the use of lyticase during the spheroplast formation from these bacteria.

## INTRODUCTION

*Mycobacterium tuberculosis* is the leading cause of adult mortality due to a single infectious agent all over the world (1). Despite new achievements in new molecular techniques for sub-specific differentiation of micro-organisms, pulse field gel electrophoresis (PFGE) still has preserved itself as one of the key tools in molecular epidemiology of infectious diseases (2). Application of this technique for genetic characterization of *Mycobacterium tuberculosis* strains can facilitate outbreak investigation and in differentiating recent transmissions from reactivation of old infections (3). The technique was previously used to differentiate the *M. tuberculosis* strains isolated from patients in Australia with sensitivity close to the standard method of RFLP using *IS*6110 as the probe (4, 5). However, controversy remains in using PFGE since it demands particular techniques to remove mycobacterial cell wall. This is the limiting reason for few numbers of papers documenting the PFGE typing of *M. tuberculosis* in the literature. Different methods have been used to resolve this problem including addition of antibiotics such as ampicillin and cycloserine in log phase growth (6), enzymatic removal of cell wall was with lyticase (4) and chemical lysis using detergents (7, 8).

In this study, we explain a new method for extraction of whole intact DNA from *M. tuberculosis* which can be used for PFGE to reduce the cost of reagents and time.

The bacterial strains used in this study were isolated from clinical specimens. *M. tuberculosis* H37RV and BCG were used as controls. We used lambda ladder as marker.

## MATERIALS AND METHODS

Bacterial cells were harvested from the slants of Lowenstein Jensen medium and heat killed at 85°C for 30 minutes. From this suspension, 350 μl was transferred into 1.5 ml micro-centrifuge tube and the cells were spun at 12,000 rpm for 5 min. The supernatant was removed and the pellet was re-suspended in 300 μl TEN buffer (100 mM EDTA, 0.15 M NaCl, 100 mM Tris pH 7.5) and centrifuged at 4500 rpm for 10 minutes. Subsequently, the sediments were suspended in 150 μl of EC buffer (100 mM EDTA, 1M NaCl, 6 mM Tris-HCl (pH 7.6), 0.2% deoxycholate–0.5% Sarkosyl and 0.5% Brij-58). The suspension was heated in boiling water for 15 minutes before mixing it with an equal volume of 2% low melting point agarose (LMP). After boiling, lysozyme (25 mg/ml) was added to the suspension (25 mg/ml). The LMP agarose was prepared in EC buffer and placed in a 56°C water bath until use. The mixture was poured into plug molds. The plugs were solidified for 5 min at 4°C. They were then transferred into 50ml falcon tubes containing 10 ml Lysis buffer I (0.5 mM EDTA, 10% Sarkosyl) and then placed in 37°C water bath overnight with gentle shaking. The plugs were transferred to 9800 μl of lysis buffer II (0.5 mM EDTA, 1% Sarkosyl) plus 200 μl of 20 mg/ml proteinase K (give the final concentration of proteinase K 0.4 mg/ml) and incubated in water bath at 50°C for 24 h with gentle shaking. Washing the plugs was done three times with 10 ml of TE buffer for 30 min. The restriction digestion step was done by adding 30 Units of *Dra*I enzymes for 16 hours incubation at 37°C after 30 min preincubation at 30°C.

The plugs were placed in wells of 1.0% agarose gels (Bio-Rad) made with 0.5X TBE, sealing with the same agarose. Restriction fragments of DNA are separated in 1% agarose in 0.5X TBE using clamped homogeneous electric fields (CHEF DRIII, Bio-Rad). The electrophoretic conditions were 6 V/cm; 120° switch angle at 14°C, and a run time of 24 h divided into two different blocks. The first block had a run time of 16 h, initial switching time of 1 s, and final switching time of 15 s, whereas the second block used a switch time ramp of 60 s–70 s for 8 h. The gel was stained with 0.5 mg/ml ethidium bromide for 20 min and de-stained in water for 20 min.

## RESULTS AND CONCLUSION

**Fig. 1:**
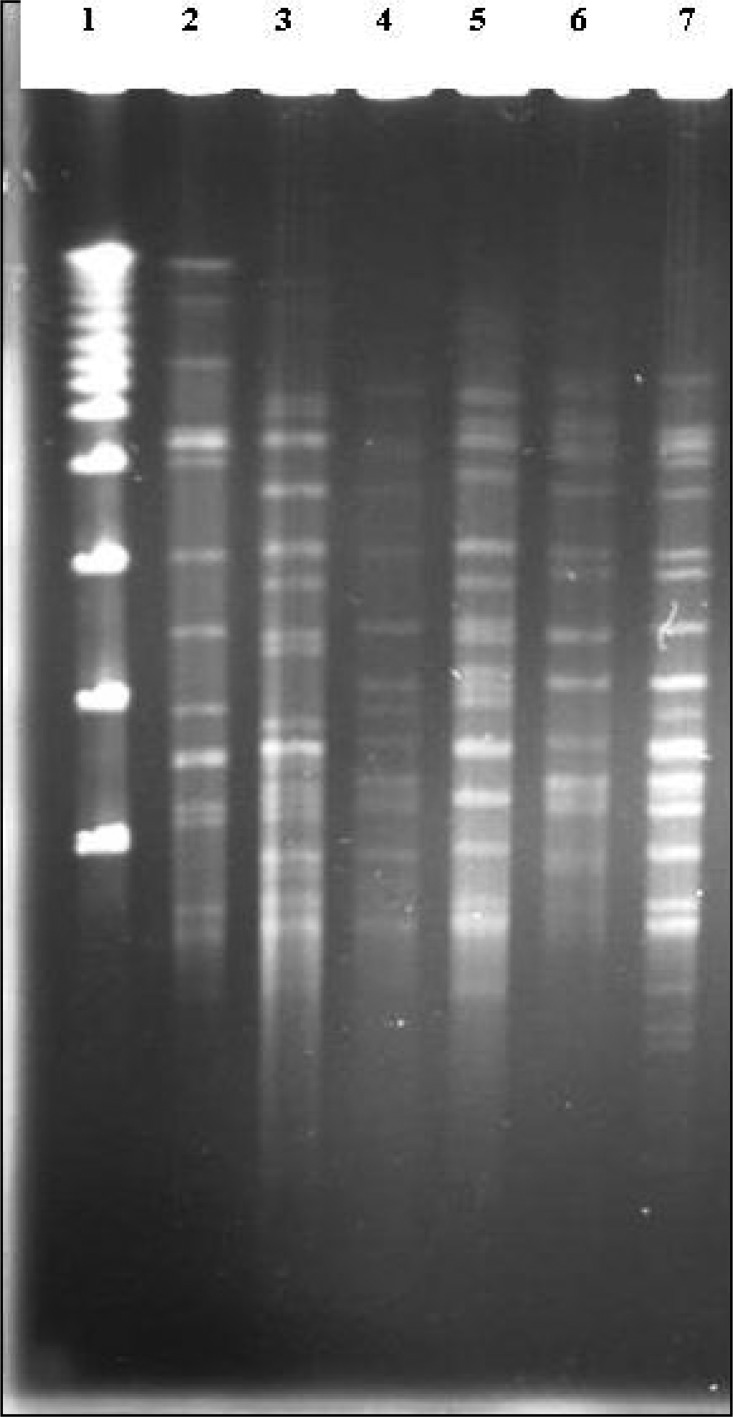
*Dra*I PFGE patterns of the *Mycobacterium tuberculosis*. Lane. 1 lambda ladder, lane 2 BCG, lane 3 *M. tuberculosis* H37RV, lanes 4-7 clinical isolates of *M. tuberculosis*.

The PFGE patterns obtained for strains of *Mycobacterium tuberculosis* are shown in Fig. 1. Pulsed–field gel electrophoresis (PFGE) has been widely used to type various microorganisms in both outbreak and population based studies and is available in many clinical laboratories as a routine typing method. Therefore, trying to modify the method especially reducing the cost of the protocol is desirable. Previously, we modified a protocol for PFGE typing genomic DNA from Staphylococcus and Enterococcus spp without using lysostaphin (9). That helped us to reduce the costs of reagents considerably since lysostaphin is an expensive reagent used in DNA extraction. In this study, we successfully developed a modified method for PFGE typing of *Mycobacterium tuberculosis complex* with elimination of lyticase which is the most expensive reagent of the protocol. The results were reproducible when further experiments were performed on additional clinical strains (Data not shown). This method for preparation of DNA for pulsed field analysis is a significant improvement over previously reported methods. Another advantage of our method is cost reduction of proteinase K which is also expensive as we used 0.4 mg/ml of this reagent in comparison with other studies that used at least 1 mg/ml (7).

In conclusion, the protocol described in this article allows cost reduction and obtained outcome is a reproducible alternative to previously published PFGE typing protocols for typing of *Mycobacterium tuberculosis* complex. We expect that the procedure will be of value during epidemiological investigations of *Mycobacterium tuberculosis* complex for comparative characterization of *Mycobacterium tuberculosis* complex isolated in different geographic loci.
